# Metagenomic Sequencing for the Diagnosis of *Plasmodium* spp. with Different Levels of Parasitemia in EDTA Blood of Malaria Patients—A Proof-of-Principle Assessment

**DOI:** 10.3390/ijms231911150

**Published:** 2022-09-22

**Authors:** Hagen Frickmann, Felix Weinreich, Ulrike Loderstädt, Sven Poppert, Egbert Tannich, Jana Bull, Bernd Kreikemeyer, Israel Barrantes

**Affiliations:** 1 Department of Microbiology and Hospital Hygiene, Bundeswehr Hospital Hamburg, 20359 Hamburg, Germany; 2 Institute for Medical Microbiology, Virology and Hygiene, University Medicine Rostock, 18057 Rostock, Germany; 3 Department of Hospital Hygiene & Infectious Diseases, University Medicine Göttingen, 37075 Goettingen, Germany; 4 Bernhard Nocht Institute for Tropical Medicine Hamburg, 20359 Hamburg, Germany; 5 Research Group Translational Bioinformatics, Institute for Biostatistics and Informatics in Medicine und Aging Research, University Medicine Rostock, 18057 Rostock, Germany

**Keywords:** *Plasmodium falciparum*, *Plasmodium malariae*, *Plasmodium vivax*, *Plasmodium ovale* complex, next generation sequencing, genomics, returnee, tropics, mixed infection

## Abstract

Molecular diagnostic approaches are increasingly included in the diagnostic workup and even in the primary diagnosis of malaria in non-endemic settings, where it is difficult to maintain skillful microscopic malaria detection due to the rarity of the disease. Pathogen-specific nucleic acid amplification, however, bears the risk of overlooking other pathogens associated with febrile illness in returnees from the tropics. Here, we assessed the discriminatory potential of metagenomic sequencing for the identification of different *Plasmodium* species with various parasitemia in EDTA blood of malaria patients. Overall, the proportion of *Plasmodium* spp.-specific sequence reads in the assessed samples showed a robust positive correlation with parasitemia (Spearman r = 0.7307, *p* = 0.0001) and a robust negative correlation with cycle threshold (Ct) values of genus-specific real-time PCR (Spearman r = −0.8626, *p* ≤ 0.0001). Depending on the applied bioinformatic algorithm, discrimination on species level was successful in 50% (11/22) to 63.6% (14/22) instances. Limiting factors for the discrimination on species level were very low parasitemia, species-depending lacking availability of reliable reference genomes, and mixed infections with high variance of the proportion of the infecting species. In summary, metagenomic sequencing as performed in this study is suitable for the detection of malaria in human blood samples, but the diagnostic detection limit for a reliable discrimination on species level remains higher than for competing diagnostic approaches like microscopy and PCR.

## 1. Introduction

Falciparum malaria is the quantitatively most important differential diagnosis in febrile travelers returning from Sub-Saharan Africa [1,2]. However, overall rare occurrence in non-endemic settings makes it difficult to maintain the training of laboratory personnel in skillful malaria microscopy as suggested by Giemsa more than 100 years ago [3] with positive slides apart from reference centers, resulting in a need for less investigator-dependent, standardizable diagnostic approaches. Molecular diagnostic techniques like real-time PCR (polymerase chain reaction) and LAMP (loop-mediated isothermal amplification) are associated with a higher sensitivity compared to microscopy even if performed in a reference center [4]. If performed from capillary blood, acceptable correlation of real-time PCR-based semi-quantification and microscopic quantification in line with the World Health Organization (WHO) standards can be achieved as well [5]. Further, species-specific real-time PCR outperforms traditional microscopy regarding the identification of mixed infections [6,7] as well as regarding the discrimination of morphologically similar parasites like *Plasmodium vivax* and *Plasmodium ovale* complex [8,9] or even of *Plasmodium ovale curtisi* and *Plasmodium ovale wallikeri* within the *P. ovale* complex [10,11,12,13]. Further, due to the superior sensitivity of the molecular test assays [14,15,16], they are of importance for the detection of reservoirs with low parasite density [17,18,19] or even submicroscopic infections [20] in the course of eradication programs [18,21,22].

In spite of such advantages of molecular malaria diagnosis, this diagnostic approach has a number of disadvantages as well. The main disadvantage of the diagnostic application of oligonucleotide-primed nucleic acid amplification techniques like real-time PCR or LAMP is the fact that they allow a targeted assessment only. So, only pathogens covered by the oligonucleotides of the assays can be detected, while other pathogens, which might have been also detected in case of microscopic assessment of stained slides, necessarily go undetected [23,24]. Although multiplexing partly compensates for these disadvantages, intrinsic technical features of the amplification techniques limit the quantitative dimension of multiplexing options. For a less specific and thus broader diagnostic screening, alternative molecular diagnostic strategies are therefore desirable.

Metagenomic sequencing is such a broad-spectrum molecular diagnostic approach [25,26,27,28,29,30,31,32,33] and has already been successfully applied for the diagnostic demonstration of *Plasmodium* spp. sequences in primary diagnostic sample materials and in the tissue of patients and even of Egyptian mummies [34,35,36,37]. As next-generation-sequencing (NGS) gets increasingly affordable [23,38] and thus more realistically applicable for diagnostic purposes, it is worth further assessing its diagnostic accuracy and potentials.

Adding to previously published work on NGS-based diagnosis of malaria [34,35,36,37], the aim of the study is a differentiated assessment of metagenomic detection of *Plasmodium* spp. in human EDTA (ethylenediaminetetraacetic acid) blood for diagnostic purposes in a standardized way. To do so, nucleic acid extractions from well characterized samples of defined malaria patients infected with different *Plasmodium* spp. with and without mixed infection with different levels of parasitemia next to a negative control obtained from a patient with a non-*Plasmodium* bloodborne parasitic infection were subjected to metagenomic NGS analysis. By doing so, the diagnostic accuracy of the approach for the diagnosis of malaria was studied in a proof-of-principle assessment.

## 2. Results

### 2.1. NGS Results and Correlation of Reads Assigned to Plasmodium spp. with Microscopically Assessed Parasitemia and Plasmodium Genus-Specific Real-Time PCR-Based Semi-Quantification

**Figure 1:**
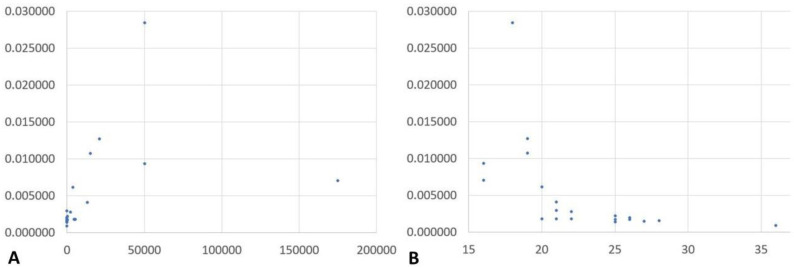
Scatter diagrams indicating the matching of the recorded reads assigned to *Plasmodium* spp. and the microscopically observed parasitemia (**A**) as well as the cycle threshold values in *Plasmodium* genus-specific real-time PCR (**B**). (**A**) *x*-axis = parasitemia in parasites per µL, *y*-axis = number of recorded reads assigned to *Plasmodium* spp. (**B**) *x*-axis = cycle threshold values in *Plasmodium* genus-specific real-time PCR, *y*-axis = number of recorded reads assigned to *Plasmodium* spp.

Successful NGS runs with raw read numbers between 13.5 and 32 million were recorded for 21 samples positive for *Plasmodium* spp. DNA and a negative control from a *Schistosoma mansoni*—infected patient (Table 1). As shown in columns 2 and 3 of Table 1, a broad spectrum of parasitemia as confirmed by microscopic assessment (column two) and semi-quantification based on cycle threshold (Ct) values of real-time PCR (column three) was assessed. In the latter case, high parasitemia is indicated by low Ct values and vice versa. The proportion of reads assigned to human DNA ranged from 95.9% to 98.9%, and the proportions of reads assigned to *Plasmodium* spp. from 0.2% to 2.9%. The proportion of reads assigned to *Plasmodium* spp. was correlated with microscopically observed parasitemia as well as with measured genus-specific Ct values obtained with the RealStar Malaria PCR Kit 1.0 (altonaDiagnostics, Hamburg, Germany). A Spearman r of 0.7307 (95%-confidence interval (CI): 0.4360, 0.8839) was calculated indicating a positive correlation of this proportion with parasitemia (*p* = 0.0001), while a negative correlation (*p* ≤ 0.0001) with a Spearman r of −0.8626 (95%-CI −0.9461, −0.6717) was calculated for the correlation with the Ct values. Scatter diagrams showing the distribution of the recorded values are provided in Figure 1. Even in the negative control sample, however, a low proportion of 0.2% reads was assigned to *Plasmodium* spp., defining an expected range of unreliable assignments. Similar proportions were observed in various samples with low parasitemia close to the microscopic detection threshold of <50 parasites/µL.

### 2.2. Matching of the Diagnostic Results on Species Level Based on the Diagnostic Reference Approach and Bioinformatic Assessments Based on Kraken, Bracken and Pavian

Concerning the identification of *Plasmodium* spp. on species level in the sample materials, the Kraken and the Bracken algorithm showed similar performance with 50% (11/22) correct identifications each as defined by the highest number of reads assigned to the respective species (Table 2). For both algorithms, the correctly and falsely assigned samples were identical. In detail, the algorithms correctly identified 2/4 *Plasmodium falciparum samples* (parasitemia range: 50.000–175.000/µL), 4/5 *P. vivax* samples (parasitemia range: 2.400–50.000/µL), 5/5 *P. ovale curtisi*/complex samples (parasitemia range: 50–20.920/µL), 0/4 *Plasmodium malariae* samples and 0/3 samples with mixed infections comprising *P. falciparum* and *P. malariae*. All 11 falsely assigned samples including the negative control samples were assigned to *P. ovale wallikeri*. Parasitemia of the falsely assigned samples of malaria patients were 50/µL as well as below the microscopic detection threshold for the two missed *P. falciparum* samples, 272/µL for the single missed *P. vivax* sample, <50–122 µL for the four missed *P. malariae* samples and 50–5240/µL for the three misidentified cases with mixed infections. Erroneous assignments were associated with assigned read numbers < 50.000, while read numbers > 50.000 were associated with assignments matching the result of the diagnostic reference approach in all observed instances.

Then applying the Pavian approach and using maximum z-scores as identifiers, the matching of the results of the diagnostic reference assessments and the NGS approach could be increased to 63.6% (14/22). In detail, the Pavian approach led to additional correct assignments of the two *P. falciparum* samples with parasitemia close to the microscopic detection limit, the *P. vivax* sample with 272 parasites/µL blood and the *P. malariae* sample with the highest parasitemia of 122/µL compared to the Kraken/Bracken approach. In contrast, one *P. ovale* complex sample with parasitemia at the microscopic detection limit of 50/µL, which had been correctly identified as *P. ovale wallikeri*/complex applying the Kraken/Bracken approach, was misidentified as *P. vivax* by the Pavian algorithm. Another misidentification with a species of etiological relevance for human patients comprised a sample containing 104/µL *P. malariae*, which reads were falsely assigned to *P. falciparum*. In the three samples from patients with mixed infections, at least *P. falciparum* as the quantitatively dominant species could be correctly identified, while the co-occurring *P. malariae* DNA went undetected in all three instances. The reads within the negative control sample were assigned to *Plasmodium gallinaceum*, a species without etiological relevance in human patients, thus indicating a false assignment. However, false assignments to *Plasmodium* species without etiological relevance in humans were also observed in two samples containing DNA of *P. malariae* with parasitemia below the microscopic detection threshold, leading to a false exclusion of human malaria in these two cases.

## 3. Discussion

The study was performed to assess the diagnostic reliability of a sequence-based metagenomic approach for the diagnosis of malaria from human blood samples. As suggested by previous studies [25,26,27,28,29,30,31,32,33,34,35,36,37], sequence-based malaria diagnosis from human primary sample materials is basically feasible. To evaluate the techniques’ diagnostic threshold and detection limits, difficult-to-diagnose reference materials comprising sub-microscopic parasitemia, parasitemia close to the microscopic detection limit and mixed plasmodial infections were included into the assessment.

As shown by the 11 to 14 perfectly matching results of reference testing and the diagnostic NGS approach as well as by the highly significant positive correlation of the *Plasmodium* spp.-specific NGS read proportion with parasitemia and the similarly good negative correlation with genus-specific Ct-values, hypothesis-free metagenomic diagnosis of malaria was confirmed to be feasible. This was true for several levels of parasitemia including a *P. falciparum*-positive sample below the microscopic detection threshold. Within the range of parasitemia close to the microscopic detection threshold, however, discrimination on species level becomes non-reliable. Varying proportions of the different species in mixed infections make NGS less suitable for their reliable identification. Further and discussed in more detail below, insufficient availability of quality-controlled reference genomes also limits the discriminatory potential on the species level.

In addition, some known general weaknesses of metagenomic diagnostic assays have been confirmed in the assessment as well. First, as known from the literature, the approach is quite laborious, still cost-intensive and time consuming [32], which limits its use for diagnostic purposes both in cases of medical emergencies and in the diagnostic routine. At least the diagnostic NGS algorithm assessed in this study still demands 2–3 days, which is considerably too long in comparison to the applied competitor approaches microscopy and real time PCR. Also, it requires several hours of hands-on-time and costs more than USD 1000 per sample (Table 3).

Second and also well-known from previous studies, the sensitivity of the assessment will depend on sequence depth [23], its reliability on the quality of the underlying databases and the applied diagnostic pipelines [23,37]. Third, the technique is volatile to contamination, which makes the discrimination of etiological relevant results from sample contaminations difficult to impossible if the target sequences occur in a similar or even lower frequency than co-occurring contaminating sequences [28,39]. Fourth, although NGS gets increasingly affordable [23,38] and in spite of modern USB-stick-shaped technical solutions, it is still technically demanding, which makes it less suitable for the point of care diagnosis of malaria at its present stage of technical development. Malaria diagnosis based on microscopy of thin and thick blood smear, in contrast, is still commonly performed in laboratories globally. This is in line with the fact that malaria microscopy is the diagnostic reference standard as defined by the World Health Organization (WHO), although microscopic skills are increasingly challenging to maintain even in endemic countries, because rapid diagnostic tests (RDT) for malaria are more frequently applied.

In the study provided here, the importance of the quality of the underlying database [23,37] was confirmed by the fact that reads were assigned to *Plasmodium* spp. even in a sample from a patient without malaria and that various misidentifications on species level occurred. Further, the depth of sequencing remains an issue [23], resulting in cases of failed or non-unambiguous species discrimination in case of parasitemia below as well as close to the diagnostic detection threshold of microscopy. The same will generally apply if the number of generated sequence reads is too low for technical reasons or due to reduced sample quality. Unfortunately, the discriminatory potential of metagenomic NGS for the diagnosis and differentiation of plasmodiae on species level was found to be inferior to the microscopic and PCR-based detection threshold for the diagnosis of malaria. On genus level, detection of reads assigned to *Plasmodium* spp. was difficult to interpret in samples with low parasitemia, because the number of recorded plasmodial reads was similar like observed in a negative control sample in this study. Of note, high variations in the proportions of different plasmodial species in case of plasmodial mixed infections made NGS poorly suitable for the identification of such mixed infections, while the z-score based approach at least allowed the identification of *P. falciparum* sequences in these cases.

Regarding the applied assessment algorithms, and since certain *Plasmodium* genomes contain sequences from host and other genomes (e.g., up to 60% of the *Plasmodium yoelli* genome identified as contaminants [40]), we first removed all contaminant sequences from the *Plasmodium* genomes present in the Ensembl database. For both the removal of contaminants and later for the taxonomic assignment of the metagenomic reads, we employed Kraken because this program is able to work in prokaryotic and eukaryotic genomes simultaneously, and it has shown an overall better performance than existing tools built for the same purposes [41,42]. Kraken aligns *k*-mers (sequence fragments of length *k*) from sequence reads to databases containing genomes with their respective taxonomic information. Reads that map to common regions in different genomes are assigned by Kraken to their lowest common ancestor (LCA). As a result, many reads are not attributed at the species level and hence Kraken assignments cannot be directly interpreted as species estimates [43]. Still, Kraken reports much more accurate results than similar tools upon normalization [44]. Therefore, we further processed the results from Kraken for abundances separately with Bracken [43] and Pavian [45]. Bracken calculates species abundances in metagenomic samples by probabilistically re-distributing reads in the taxonomic tree, i.e., reads are assigned above the species level are incorporated into the species level, while reads allocated at the strain level are re-distributed upwards to its corresponding parent species level [43]. Similarly, we used Pavian, an online tool that allows the visual inspection of Kraken results, as well as providing different transformations of the Kraken output data, such as the z-score of the assigned reads [45]. Both Bracken and Pavian have been employed in the diagnostics of infectious diseases through metagenomic assignment, and the results from Kraken were particularly improved upon using the z-score, as implemented in Pavian [46]. In this regard, since both Bracken and Pavian work on Kraken results, and these in turn are affected by the sequencing quality and coverage, we expect that the results from the metagenomic methods presented here will be greatly improved whenever the genomes from the target databases are less prone to contaminant sequences, and sequencing replicates and higher sequencing coverages are available for the clinical samples.

The study has a number of limitations. First, only a small number of samples could be included due to funding restrictions. Second, no patient-specific clinical details could be provided in line with the ethical demands allowing the use of anonymized residual sample materials for the diagnostic evaluation only. Third, it remains unclear if better matching might have resulted from the use of a more comprehensive database containing quality-controlled genomes of organisms other than just *Homo sapiens* and *Plasmodium* spp. However, such a comprehensive approach would have been beyond the scope of this proof-of-principle assessment with limited available resources.

## 4. Materials and Methods

### 4.1. Study Design and Sample Materials

The comparative study evaluating the metagenomic diagnosis of malaria in human EDTA blood was conducted in a single-blinded way, meaning that the study partner performing the NGS analyses did not know the microscopic and molecular diagnostic results when the assessments were performed. The workflow of the assessment is provided below in Table 4.

In detail, a total of 22 samples was assessed, one sample per NGS run was investigated. Among the samples, 21 out of 22 had been found to be positive for malaria due to *P. falciparum*, *P. malariae*, *P. vivax* or *P. ovale* complex comprising *P. ovale curtisi* and *P. ovale wallikeri* in mono-infections or mixed infections as confirmed by microscopy as recommended by WHO as well as molecular diagnostic approaches as described previously [4]. *P. knowlesi*-positive samples could not be included, because such residual materials were not available. In detail, the molecular diagnoses comprised genus-specific malaria detection applying commercial LAMP (Alethia Malaria, Meridian Bioscience Inc., Cincinnati, OH, USA) [47] and real-time PCR (RealStar Malaria PCR Kit 1.0, altona Diagnostics, Hamburg, Germany) [8] as well as species-specific SybrGreen-based real-time in-house PCR [48,49] and commercial species-specific real-time PCR (FTD Malaria differentiation, Fast Track Diagnostics, Sliema, Malta; RealStar Malaria S&T Kit 1.0, altona Diagnostics, Hamburg, Germany) [8]. Samples positive for *P. ovale* complex were differentiated into *P. ovale curtisi* and *P. ovale wallikeri* by in-house real-time PCR as described elsewhere [10,11,12,13]. Of note, one of the reference samples had been repeatedly misidentified as *P. vivax* by microscopy while only PCR revealed the identity as *P. ovale* complex [8]. Microscopic quantification as well as semi-quantification based on recorded cycle-threshold (Ct) values of real-time PCR had been conducted for the characterization of the reference materials. The samples were chosen by ensuring that specimens were included with a spectrum of the parasitemia ranging from submicroscopic infections (<50 parasites/µL) till one-digit percentages of infected erythrocytes. This was done in order to define the detection thresholds of the metagenomic NGS approach for identifications on genus level, on species level as well as for the discrimination of mono-infections and mixed infections. Finally, an EDTA blood sample from a patient with early schistosomiasis (Katayama fever) caused by *S. masoni* as confirmed by in-house serum duplex real-time PCR targeting *Schistosoma haematobium* complex and *S. mansoni* complex [50] was included as a negative control. Patient specific data could not be included in the assessment, because complete anonymization of the investigated sample materials was an ethical requirement as demanded by the ethics committee for the described diagnostic test assessment. This is an admitted deviation from the STARD (Standards for Reporting Diagnostic Accuracy) criteria [51].

### 4.2. Metagenomic Next Generation Sequencing

Nucleic acid sequences used for metagenomic NGS had been extracted applying the EZ1 DNA Blood 200 µL Kit using automated EZ1 nucleic acid extractors (Qiagen, Hilden, Germany) as described by the manufacturer and detailed elsewhere [52]. Prior to the assessments, the samples had been stored frozen at −80 °C. Metagenomic unbiased NGS sequencing of the DNA elements in the samples was conducted by an experienced medical-laboratory assistant using a MiSeq system (Illumina, San Diego, CA, USA) applying the protocols provided by the manufacturer. No target DNA enrichment or human DNA depletion was done. In short, DNA libraries were prepared with TruSeq^®^ Nano DNA Sample Preparation kits (Illumina) using the low sample (LS) protocol. Thereby, 100 ng of each genomic DNA from the assessed samples was fragmented using Adaptive Focused Acoustics^TM^ Technology (Covaris, Inc., Woburn, MA, USA) with a Covaris M220 applying settings for fragment sizes in the 350 bp range (duty factor 20%, peak incident power 50 W, cycle per burst 200, duration 65 s, at a temperature of 65 °C). The chromosomal DNA fragments were cleaned up with bead technology. End repair was done in line with the TruSeq protocols. Additional clean-up and size selection was conducted with bead technology. 3’-ends were subsequently adenylated, Illumina adapters were ligated and the DNA fragments were enriched. An Agilent DNA 7500 kit (Agilent Technologies, Inc., Santa Clara, CA, USA) was applied for quality control and for the confirmation of the intended fragment size after the application of the Covaris M220 fragmentation protocol and after Illumina adapter ligation. Visualization of clearly defined peaks in the expected size range was considered as proof of successful DNA fragmentation and adapter ligation. No concentration calculation by integrating the area under the peak was conducted, because this was considered as insufficiently sensitive for sequencing purposes. Instead of this, library DNA concentration measurements were performed using Qubit dsDNA BR assay kits (Thermo Fisher Scientific, Waltham, MA, USA), before the sequencing cells were loaded. After this assessment, each individual library was adjusted to a 4 nmol/L stock solution and from these stocks, 6 pmol was used for each individual sequence run. Sequencing was conducted as Reagent Kit MiSeq^®^ v3 (600 cycle) runs (Illumina). Each sample was assessed with a complete v3 run.

### 4.3. Bioinformatic Sequence Analysis and Statistics

The bioinformatic analysis consisted of three steps: First, cleaning the *Plasmodium* genomes from contaminants; then building a custom database consisting of *Plasmodium* and human genomes; and finally assigning the clinical metagenomic sequencing reads to the corresponding *Plasmodium* genomes present in the custom database. For the first part, and as certain *Plasmodium* genomes are contaminated with host and microbial sequence data [40], *Plasmodium* spp. genomes were retrieved from Ensembl [53], and cleaned from contaminants with the following protocol: (i) slicing the genome sequences into pseudoreads (length 100 bp, overlap 50 bp) with pyfasta version 0.5.2 [54] and BBtools version 38.95 [55]; and (ii) assigning these pseudoreads to non-*Plasmodium* genomes, as present in the Standard-16 Kraken database, with Kraken version 2.1.2 [56]. The Standard-16 database consists of sequences that can be considered common contaminants of human samples, such as genomes from archaea, bacterial, viral, plasmid, Univec_Core and unmasked human sequences present in the RefSeq database (retrieved from https://benlangmead.github.io/aws-indexes/k2, accessed on 19 September 2022). Pseudoreads matching these contaminant genomes from this database were discarded from the genome assemblies, as well as any fragments smaller than 100 bp [40]. Afterwards, a custom Kraken database was built with these “clean” *Plasmodium* genome assemblies together with the unmasked human genome, which was then used to assign the metagenomic reads from the EDTA blood samples with Kraken to the taxonomic levels present in this custom database. These taxonomic assignments provided by Kraken were then further processed for abundance separately with Bracken version 2.7 [43] and Pavian (package version 1.2.0 [44]). In the case of our clinical metagenomic samples, Bracken was run at the species level and using a read size of 100 bp, while for Pavian we employed the z-score over the Kraken-assigned reads. All other parameters were kept with default values for both programs. The metagenomic sequencing runs were deposited on the European Nucleotide Archive (ENA) under the accession number PRJEB55471.

Correlations between the proportion of *Plasmodium* spp.-specific reads and microscopically observed parasitemia as well as measured genus-specific cycle threshold (Ct) values obtained with the RealStar Malaria PCR Kit 1.0 (altonaDiagnostics, Hamburg, Germany) were calculated applying Spearman rank correlation testing, for which no Gaussian distribution needs to be assumed. The calculation was conducted applying the software GraphPad Instat, version 3.06 (GraphPad Software Inc., La Jolla, CA, USA).

### 4.4. Ethical Clearance

Ethical clearance for the anonymized use of residual sample materials for test evaluation purposes without requirement of informed consent was provided by the medical association of Hamburg, Germany (reference number: WF-011/19, obtained on 11 March 2019) in line with National German laws. The study was performed according to the Declaration of Helsinki and its amendments.

## 5. Conclusions

The assessment proved the general suitability of the applied metagenomic diagnostic approach for the diagnosis of malaria from human blood. The limitations as detailed in the discussion, however, still leave considerable room for improvement of the technique before application in the diagnostic routine or even in case of emergency situations can be recommended.

## Figures and Tables

**Table 1:** Read assignment of the next generation sequencing (NGS) runs.

Sample Code	Parasitemia with *Plasmodium* spp. as Assessed by Microscopy	Cycle Threshold Value in Genus Species PCR for *Plasmodium* spp.	Number of Total Reads	Number of Reads Assigned to *Homo sapiens*, in Brackets: Percentage of Total Reads	Number of Reads Assigned to *Plasmodium* spp., in Brackets: Percentage of Total Reads
N.C.	0/µL	n.a.	25,002,603	24,515,911 (98.05%)	53,342 (0.21%)
D016	50.000/µL	16	13,682,913	13,303,308 (97.23%)	129,035 (0.94%)
D169	<50/µL	28	22,763,368	22,249,906 (97.74%)	37,024 (0.16%)
D170	272/µL	25	23,842,401	23,364,531 (98.00%)	52,209 (0.22%)
D178	175.000/µL	16	27,137,956	26,494,085 (97.63%)	192,375 (0.71%)
D020	50/µL	26	20,402,946	20,018,621 (98.12%)	40,501 (0.20%)
D216	50.000/µL	18	16,695,865	16,007,009 (95.87%)	475,761 (2.85%)
D225	14.920/µL	19	26,345,466	25,669,538 (97.43%)	283,245 (1.08%)
D234	4.000/µL	20	17,687,032	17,412,071 (98.45%)	110,231 (0.62%)
D270	<50/µL	27	17,725,864	17,532,908 (98.91%)	26,938 (0.15%)
D272	104/µL	26	24,323,327	23,844,801 (98.03%)	40,256 (0.17%)
D282	122/µL	25	15,819,159	15,634,285 (98.83%)	26,959 (0.17%)
D293	5.000/µL	20	19,060,562	18,855,573 (98.92%)	34,745 (0.18%)
D302	50/µL	36	20,613,039	20,353,551 (98.74%)	19,257 (0.09%)
D417	13.000/µL	21	22,729,535	22,409,043 (98.59%)	93,498 (0.41%)
D465	<50/µL	negative ^	23,225,621	22,888,629 (98.55%)	38,203 (0.16%)
D503	50/µL	21	25,935,179	25,647,132 (98.89%)	78,501 (0.30%)
D558	2.400/µL	22	24,659,561	24,346,841 (98.73%)	69,882 (0.28%)
D567 *	4.600/µL	22	28,609,738	27,803,374 (97.18%)	50,884 (0.18%)
D570	5.240/µL	21	18,913,510	18,713,593 (98.94%)	34,637 (0.18%)
D583 *	50/µL	25	31,978,731	31,467,597 (98.40%)	43,995 (0.14%)
D747 °	20.920/µL	19	23,097,171	22,652,992 (98.08%)	293,161 (1.27%)

N.C. = negative control sample. * Samples from the same patient at different time points. ° Sample had initially been microscopically misidentified as *P. vivax* in microscopy. n.a. = not applicable. ^ *Plasmodium malariae* DNA had been detected by a species-specific PCR assay only.

**Table 2:** Assignment of *Plasmodium* spp. by the Kraken, Bracken and Pavian assessments.

Sample Code	Species According to the Reference Diagnostic Approach	*Plasmodium* Species Applying the Kraken Approach (Number of Assigned Reads)	*Plasmodium* Species Applying the Bracken Approach (Number of Assigned Reads)	*Plasmodium* Species Applying the Pavian Approach (Calculated Z-Score)
N.C.	*Schistosoma mansoni*	*Plasmodium ovale wallikeri* (46,154)	*Plasmodium ovale* complex (48,062)	*Plasmodium gallinaceum* (3.4)
D016	*Plasmodium falciparum*	*P. falciparum* (65,171)	*P. falciparum* (103,363)	*P. falciparum* (21,980.0)
D169	*P. falciparum*	*P. ovale wallikeri* (29,240)	*P. ovale* complex (30,597)	*P. falciparum* (10.5)
D170	*Plasmodium vivax*	*P. ovale wallikeri* (36,296)	*P. ovale* complex (36,872)	*P. vivax* (4.3)
D178	*P. falciparum*	*P. falciparum* (105,104)	*P. falciparum* (168,368)	*P. falciparum* (35,440.0)
D020	*Plasmodium falciparum*	*P. ovale wallikeri* (30,068)	*P. ovale* complex (31,501)	*P. vivax* (52.3)
D216	*P. vivax*	*P. vixax* (380,816)	*P. vivax* (457,652)	*P. vivax* (374.8)
D225	*P. vivax*	*P. vivax* (222,565)	*P. vivax* (259,235)	*P. vivax* (218.4)
D234	*P. vivax*	*P. vivax* (82,354)	*P. vivax* (98,480)	*P. vivax* (79.9)
D270	*Plasmodium malariae*	*P. ovale wallikeri* (21,138)	*P. ovale* complex (21,682)	*Plasmodium relictum* (1.0)
D272	*P. malariae*	*P. ovale wallikeri* (32,768)	*P. ovale* complex (34,566)	*P. falciparum* (3.7)
D282	*P. malariae*	*P. ovale wallikeri* (20,891)	*P. ovale* complex (21,482)	*P. malariae* (0.3)
D293	*P. ovale curtisi*	*P. ovale curtisi* (18,404)	*P. ovale* complex (31,708)	*P. ovale* complex (128.4)
D302	*P. ovale* complex	*P. ovale wallikeri* (13,743)	*P. ovale* complex (13,820)	*P. vivax* (2.0)
D417	*Plasmodium ovale curtisi*	*P. ovale curtisi* (18,404)	*P. ovale* complex (90,625)	*Plasmodium ovale* complex (404.5)
D465	*P. malariae*	*P. ovale wallikeri* (28,460)	*P. ovale* complex (30,082)	*Plasmodium* spp. (1.0)
D503	*P. ovale curtisi*	*P. ovale curtisi* (55,869)	*P. ovale* complex (77,118)	*P. ovale* complex (390.5)
D558	*P. vivax*	*P. vivax* (35,625)	*P. vivax* (43,473)	*P. vivax* (33.7)
D567 *	*P. falciparum* & *P. vivax*	*P. ovale wallikeri* (29,901)	*P. ovale* complex (31,972)	*P. falciparum* (390.5)
D570	*P. falciparum* & *P. vivax*	*P. ovale wallikeri* (23,969)	*P. ovale* complex (24,521)	*P. falciparum* (2241.0)
D583 *	*P. falciparum* & *P. vivax*	*P. ovale wallikeri* (33,528)	*P. ovale* complex (35,859)	*P. falciparum* (136.2)
D747 °	*P. ovale curtisi*	*P. ovale curtisi* (263,024)	*P. ovale* complex (291,205)	*P. ovale* complex (1648.0)

N.C. = negative control sample. * Samples from the same patient at different time points. ° Sample had initially been microscopically misidentified as *P. vivax* in microscopy.

**Table 3:** Time-to-result, hands-on-time and material costs of the diagnostic approaches for the diagnosis of malaria which were compared in this study.

	Microscopy	Traditional Molecular Diagnostic Approaches (e.g., Real-Time PCR, Loop-Mediated Isothermal Amplification)	Diagnostic Application of the Described Next Generation Sequencing Approach
Time required for the diagnostic workflow	About 1 h	1 to few hours	2–3 days
Hands-on-time	About 1 h	Few minutes to 1 h	Several hours
Reagent costs per sample	Less than 1 US dollar	Less than 100 US dollars	More than 1000 US dollars

**Table 4:** Summarized workflow of the assessment.

Inclusion of residual sample materials of 21 serum samples found to be positive for malaria in previous test evaluation approaches [4] comprising microscopic assessment and species-specific real-time PCR covering various plasmodial species and a broad range of parasitemia Inclusion of a single residual sample material of a serum from a patient suffering from an infection with the non-malaria blood parasite *Schistosoma mansoni* as a negative control
↓
All 22 samples subjected to MiSeq^®^ (Illumina, San Diego, CA, USA)-based next generation sequencing
↓
Assessment of the sequence reads applying 3 different bioinformatic algorithms for the identification of plasmodial target sequences
↓
Comparison of the results obtained with the applied bioinformatic analyses of the sequences to the results obtained with the composite reference standard based on real-time PCR and microscopy

## Data Availability

All relevant data are provided within the manuscript and its appendix or are accessible via the links provided in the text. The Illumina sequencing data was deposited on the European Nucleotide Archive (ENA) under the project accession number PRJEB55471.
